# Seeing is Believing but is Hearing? Comparing Audio and Video Communication for Young Children

**DOI:** 10.3389/fpsyg.2013.00064

**Published:** 2013-02-28

**Authors:** Joanne Tarasuik, Roslyn Galligan, Jordy Kaufman

**Affiliations:** ^1^The Brain and Psychological Sciences Research Centre, Swinburne University of TechnologyMelbourne, VIC, Australia

**Keywords:** child development, communication, separation, computer mediated communication, telephone, video, verbal, non-verbal

## Abstract

Video communication has been shown to create a sense of proximity between young children and parents. To determine if video affords a stronger sense of proximity and engagement than a traditional telephone, the current experiment employed a Separation and Reunion Paradigm with either a video-link or an audio-link available to the separated dyad. Results revealed that during the separation with a video-link, more children remained content to be physically alone than during the audio-link, children played more and displayed more positive affect. This is the first empirical demonstration that video provides a stronger sense of proximity and enjoyment for young children than audio, suggesting that video is a more appropriate medium to meaningfully connect children to relatives during geographical separation.

## Introduction

Since its invention in the late 1800s the telephone has played an important role in communication. Today however, internet infrastructure has enhanced communication options such that people are no longer limited to only talking to each other, but can now simultaneously see each other. Video communication has become increasingly popular as a means of maintaining relationships when actual face-to-face contact is not possible (Symantec Corporation, 2009), particularly with children (Harmon, 2008; Taub, 2009). Whilst our previous research has established that video communication provides young children with a sense of proximity and security with an absent parent (Tarasuik et al., 2011), it remains underdetermined whether the sense of security a child feels with a video-enabled virtual parental presence is superior to what can be attained with a more telephone-like audio communication channel.

One reason to hypothesize that video communication is the superior medium for maintaining close emotional links is that whilst young children may appear to participate in telephone communication to some extent, the complete spectrum of skills required for effective telephone communication may not develop until the early primary school years (Ballagas et al., 2009). Video communication however does not appear to require many of the demands of telephone communication. The ability to communicate verbally, for example, is a prerequisite for telephone communication given that it is ineffective until comprehensible articulation develops. Conversely, the visual cues available during video communication such as body language and facial expressions can assist an adult in interpreting the unclear speech of young children; and posture, gestures, and other body language can assist children with both expression and comprehension (Ballagas et al., 2009). Another determinant of effective telephone communication is decontextualized language (Ricard and Snow, 1990), yet young children’s language is very much contextualized in the here and now – so that what they talk about is something present and usually seen or able to be appreciated by both the child speaker and the adult listener. Without the appropriate visual cues, a child is not able to fill in the contextual details verbally, nor can they adequately understand decontextualized speech. Furthermore, when solely reliant upon verbal communication, children under 4 years of age generally lack important response discourse skills and rarely initiate conversation (Bordeaux and Willbrand, 1987).

In addition to these cognitive and linguistic demands of telephone communication, young children often struggle to position a telephone appropriately to both speak into the mouthpiece and hold the speaker next to their ear so able to concurrently contribute and listen to a conversation. Such constraints to telephone discourse, may significantly limit the gratification and benefits to audio-only communication with young children. Children up to 9 years of age for example, have difficulty paying attention during telephone conversations (Ballagas et al., 2009). Video communication has the potential to overcome most, if not all the problems that young children experience with telephone communication. Adults have indicated that they enjoy the richness of the interaction permitted by video communication (Symantec Corporation, 2009), particularly when conversing with children (Harmon, 2008; Taub, 2009). For children themselves, relative to a telephone call, the difference may be exponentially richer, especially for infants and toddlers with limited or no verbal language skills.

In our previous research (Tarasuik et al., 2011) we established that when young children were briefly physically separated from their parent but connected through a video-link, they showed fewer signs of separation anxiety than when they experienced separation without this link. Provision of the video-link enabled the children to maintain feelings of proximity and security. However, because video and audio were both part of this video-link, we can only hypothesize about whether the video had an effect above and beyond an audio-only link. Thus, the current experiment aimed to establish if video is the essential element in contributing to an effective parent-child link during a separation. As in Tarasuik et al. (2011) a modification of the separation and reunion paradigm was employed to compare the value of video vs. audio-only interaction for young children. The age range of 18–36 months was selected as to best expand upon our previous research comparing virtual parental presence, physical presence, and complete parental absence (Tarasuik et al., 2011), as the results were strongest within this age range, and Attachment behaviors are known to occur with this age range (e.g., The MacArthur Preschool Strange Situation; Cassidy and Marvin, 1992).

Given the additional richness of video, the central hypothesis of the current experiment was that children would experience a stronger sense of proximity and engagement with their parent during a video-link than an audio-link. Accordingly, children were expected to remain content to be alone in an unfamiliar environment for a longer period of time when we provided a video-link to the parent than when we provided an audio-only link. Additionally, it was hypothesized that attachment behavior that would be expected to intensively activate during a separation (Ainsworth et al., 1971) would be attenuated more by a video-link than an audio-link. As a result, during a video-link, children’s affect was expected to resemble their affect when the parent was physically present more so than during the audio-link.

## Materials and Methods

### Apparatus and materials

The Section “Materials and Methods” used were based on those used by Tarasuik et al. (2011). The experiment was conducted in two adjoining lab spaces consisting of a child play-room and a parent computer room. The child play-room was 175 cm × 300 cm which contained a couch, age-appropriate toys (including a drawing easel and markers, blocks, a train set, and soft toys), and a computer monitor for communicating with a parent in the adjoining room. Three cameras were positioned within the play-room to capture children’s behavior including any interactions with their parent. The computer room contained a desk and a computer on which the parent could view and/or communicate with their child in the play-room. The video-links were established and recorded using commercially available video communication application software. The picture-in-picture feature was activated, so during the video-link the play-room monitor presented a full-screen image of the parent, with an insert of the play-room footage presented in the top right corner of the monitor, and the reverse was presented on the parent’s computer monitor.

A parent completed a brief demographic questionnaire about his/her child, and to establish if the child was securely attached they also completed the Attachment Q-Set (AQS) questionnaire which was based on the AQS (Waters, 1995). The original AQS involved the observation of attachment-related behaviors during which a child was positioned on a continuum from insecure to secure using a Q sort to make this judgment (Waters, 1995). We used a modified AQS questionnaire that asked parents to rate each of the statements from the AQS cards, on a five point Likert scale to indicate the degree to which the statement was true of the child with responses of −1, −0.5, 0, +0.5, +1 where −1 indicated “never true,” and +1 “always true.” The scores of the AQS items assessing secure attachment (items 1, 4, 11, 18, 28, 44, 47, 60, 64, and 70) were examined to compute a mean secure score. Comparable to the AQS 3.0 (Waters, 1995), the possible score range was from −1 to +1 with positive scores indicative of a secure child. Only children with a score greater than zero (i.e., indicative of a securely attached child) were included in the experiment, excluding one child. The findings of the current study are therefore likely reflective of securely attached children.

### Participants

Participants included in the analysis were 25 (male = 14, female = 11) children aged 25.0–42.5 months (*M* = 32.6, SD = 5.36). Nine children were aged 2–2.5 years; nine were aged 2.5–3 years; and seven were aged 3 years. The majority of children (*n* = 22) participated with their mother rather than their father. An additional five children participated but were excluded from the analysis due to technical problems with the cameras. Participants were recruited through online and print advertisements as well as word-of-mouth referrals.

### Procedure

The protocol was approved by the Swinburne University Human Research Ethics Committee. After obtaining informed consent, each parent-child dyad participated in a separation and reunion protocol, as used in Tarasuik et al. (2011). The current protocol involved a *free-play* episode followed by two separation-reunion episodes. During each separation episode the dyad were connected by either a video-link, which allowed audio and visual interaction, or an audio-only link (see Table 1). The order of these separations was counter-balanced across participants.

**Table 1:** **Description of the free-play, separation, and reunion episodes**.

Episode	Duration	Description
Free-play	10 min	The parent and child were left alone in the play-room with the only instruction to interact normally, and that the researcher would return after 10 min
Video separation	≤5 min	The researcher entered the play-room and asked the parent for assistance in another room. The parent told their child that he/she would return soon and left the room. The researcher then took the parent to an adjacent room and the parent was asked to interact with the child via the video-link. The episode was terminated after 5 min, or earlier if the child showed signs of distress
Reunion 1	5 min	The parent returned to the play-room without any further instructions
Audio separation	≤5 min	Consistent with the video separation except that the parent and the child could hear but not see each other
Reunion 2	5 min	Consistent with reunion 1, after which the researcher entered the room to conclude the session

### Coding

The maximum duration coded for each episode was 300 s. Behavioral variables *play* and *interaction* were coded as present or absent in each 10-s interval of the video recordings for both the *free-play* episode and the two separation episodes (see Table 2). In line with Tarasuik et al. (2011), the *play* and *interaction* variables were examined as the ratio of the number of intervals in which the behavior was present to the total number of intervals in that episode. For example if a child remained content for 200 s, i.e., twenty 10-s intervals, and was coded as having played in 10 intervals, the play ratio was 50%. Additional analyses examining variables by the number of intervals rather than as a percentage resulted in the same effects and trends as reported in the Section “Results.”

**Table 2:** **Descriptions of variables**.

Variable	Comparing episodes	Measurement unit	Description
Contentment	Audio sep Video sep	Seconds	The period of time that the child was content to be physically alone in the play-room. Timed from the parent leaving the play-room until the child showed distress for 10 s, or attempted to leave the room. The maximum score was 300 as the maximum duration of each separation was 300 s (i.e., 5 min)
Maximum contentment	Audio sep	Yes/no	Did the child remain content for the entire 300 s?
	Video sep	
Play	Free-play Audio sep Video sep	%	The percentage of intervals that the child remained during which he/she played. Play was defined as handling or otherwise interacting with toys as well as dancing, making funny faces with the parent, admiring their block buildings, etc
Interaction	Free-play Audio sep Video sep	%	The percentage of intervals that the child remained during which he/she talked (verbally communicated to the best of the child’s ability) to the parent
Response ratio	Free-play Audio sep Video sep	%	The percentage of parent’s questions to which the child responded. Responses could be verbal, non-verbal, and/or action responses
Affect	Free-play Audio sep Video sep	Score 1–5	Mean affect across the intervals of each episode. Rated on a five point Likert scale from 1 = extreme negative emotion, to 5 = extreme positive emotion, with three the neutral rating

Cohen’s Kappa revealed substantial inter-rater reliability for play during the video (κ = 0.84, *p* < 0.001), audio (κ = 0.84, *p* < 0.001), free-play episodes (κ = 79, *p* < 0.001), and interaction during the video (κ = 0.70, *p* < 0.001), audio (κ = 0.70, *p* < 0.001), free-play episodes (κ = 0.81, *p* < 0.001). One of the coders was blind to the experimental hypotheses.

## Results

Preliminary statistical analyses indicated that children’s gender, previous video communication experience, and separation order did not have any effect on the dependent variables (*p* > 0.05 for all comparisons) and were eliminated from further analysis. Since data violated the assumptions required to perform parametric tests non-parametric tests were used.

Following Cohen’s (1988, pp. 79–81) criteria, effect sizes reported for the Wilcoxon Signed Ranks tests have been set as *r* = 0.1 < 0.3 for a small effect size, *r* = 0.3 < 0.5 for a medium effect size, and *r* = 0.5 < 1 for a large effect size.

Table 3 shows the median values of behavioral indicators for each episode, and results of statistical tests.

**Table 3:** **Medians behavioral scores across episodes and Wilcoxon signed rank test details**.

Measure	Mdn			Comparison		
	FP	V	A	
Play	100%	90%	80%	Free-play vs. video*	*p* = 0.009	*z* = −2.615
				Free-play vs. audio*	*p* < 0.001	*z* = −3.502
				Video vs. audio*	*p* = 0.005	*z* = −2.810
Interaction	57%	53%	48%	Free-play vs. video*	*p* = 0.004	*z* = −2.895
				Free-play vs. audio*	*p* = 0.007	*z* = −2.715
				Video vs. audio	*p* = 0.234	*z* = −1.189
Affect	3.17	3.22	3.00	Free-play vs. video	*p* = 0.279	*z* = −1.084
				Free-play vs. audio*	*p* = 0.001	*z* = −3.382
				Video vs. audio*	*p* = 0.022	*z* = −2.294
Response ratio	69%	66%	50%	Free-play vs. video	*p* = 1.000	*z* = 0.000
				Free-play vs. audio*	*p* = 0.022	*z* = −2.286
				Video vs. audio	*p* = 0.085	*z* = −1.720

*p < 0.05

Initial analysis of the contentment data revealed that a large proportion of participants remained content for the entire 300-s duration. Consequently, analyses were performed on the categorical variable “*maximum contentment*” defined by whether a child had or had not remained in the room for the full separation duration. McNemar tests revealed that significantly more participants achieved the *maximum contentment* duration for the *video separation* (88%) than the *audio separation* (64%) with a medium effect size. [χ^2^(1, *n* = 25) = 4.50, *p* < 0.05, Φ = 0.3, the odds ratio is 7.0].

A Friedman test comparing the percentage of intervals that children *played* showed that children *played* the most during the *free-play episode*, followed by *video separation*, and the least during the *audio separation*, χ^2^(2, *n* = 25) = 19.432, *p* < 0.001. Further planned comparisons using Wilcoxon Signed Ranks tests (see Table 3) revealed that children *played* significantly more during the *free-play episode* than both the *video and audio separations* both with a large effect size, and they *played* significantly more during the *video separation* than the *audio separation*, also with a large effect size.

**Figure 1:**
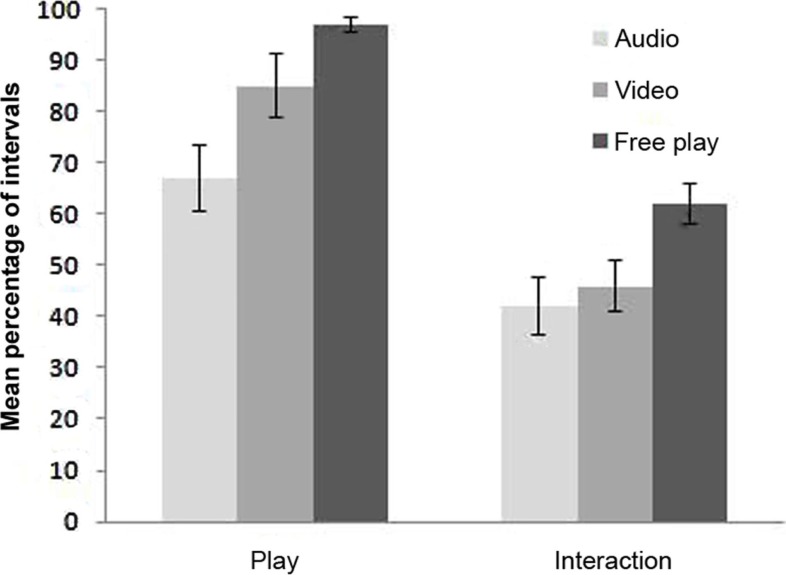
**Mean percentage of play and interaction during the free-play and separation episodes**.

A Friedman test comparing the percentage of intervals that children *interacted* revealed that children *interacted* the most during the *free-play episode* followed by the *audio* and *video separations*, χ^2^(2, *n* = 25) = 8.313, *p* = 0.016. Further planned comparisons using Wilcoxon Signed Ranks tests (see Table 3) indicated that children *interacted* significantly more during the *free-play episode* than both the *video and audio separations*, each with a large effect size, but that they *interacted* a comparable amount of time during the *video and audio separations*. The means of *play* and *interaction* across the episodes are presented as Figure 1.

A Friedman test comparing children’s *affect* revealed that affect was the most positive during the *free-play episode*, followed by the *video separation*, and the least positive during the *audio separation*, χ^2^(2, *n* = 24) = 11.293, *p* = 0.004. Further planned comparisons using Wilcoxon Signed Ranks tests (see Table 3) indicated that *affect* during the *free-play* and the *video separation* episodes was comparable, *affect* was more positive during both the *free-play* episode and the *video separation* episode than during the *audio separation* with a large and medium effect size.

To investigate the quality of interaction during the episodes, a Friedman test was performed to examine *response ratio*, which indicated that the conditions did not differ, χ^2^(2, *n* = 19) = 4.829, *p* < 0.089. Further planned comparisons using Wilcoxon Signed Ranks tests (see Table 3) indicated that *response ratio* during the *free-play episode* was comparable to the *video separation*, but that *response ratio* was significantly greater during the *free-play episode* than during the *audio separation* with a medium effect size. There was a trend for greater *response ratio* during the *video separation* than the *audio separation*, which was not significant with a two tailed test, however the hypothesis included direction, and with a one-tailed test, in the hypothesized direction the finding was significant.

## Discussion

The fundamental contribution of this research is the confirmation that, for young children, a parental presence via video provides a stronger sense of proximity than an audio-only presence. Whilst previous research has demonstrated limitations of telephone use by young children (Ballagas et al., 2009), our research demonstrates the potential of video communication to overcome most of those shortcomings. These experimental findings are informative in considering the enhanced potential of video communication to play a role in the maintenance or formation of relationship when geographical proximity is not possible.

Our conclusions are based on three measures of child behavior: contentment with physical isolation from the parent, interaction with the parent, and exploration and play, all of which are common indices of attachment security (Ainsworth et al., 1971).

With respect to our measure of child contentment during the separation episodes, a greater percentage of children were content for the entire 5-min video separation than the audio separation. This result was consistent with the results of Tarasuik et al. (2011) where more enduring contentment was evident during the video than the non-video separation. Contentment is arguably the most direct measure of how secure our participants felt when they were in the room which demonstrates that a video-link is a superior medium for young children to obtain a sense of proximity to their parent. Furthermore, given that the separations were limited to 5 min, the content participants were likely to have remained content much longer, had they been provided the opportunity.

Not only were children more willing to experience a lengthy separation when there was a video-link compared to an audio-link, their affectivity was more positive when a video-link was available. Additionally children demonstrated comparable quality of interaction with their parent during the video-link, to when the parent was physically in the room, whilst it was significantly less during the audio-link. Furthermore since the hypothesis was directional, postulating a greater response ratio during the video-link, one-tailed tests could have been reported which produced a significant difference, illustrating that the video-link was more engaging than the audio-link. Such findings are consistent with Ames et al. (2010) who concluded from their observational study comparing telephone to video communication, that engagement is one of the big advantages of video communication with children. For video communication to be used for the maintenance or formation of relationships, it is imperative that children enjoy such interaction. The finding that video communication encourages enjoyable interactions between the partners is promising as motivation to participate is fundamental in virtual presence holding any value. The reciprocal interaction during the parents’ virtual presence, akin to interaction during an actual physical presence, demonstrates that video induces a stronger quality of engagement than audio, and reveals the cogency of virtual presence relative to physical presence.

The attachment literature outlines that children use their parent as a secure base to explore the environment, and accordingly play more when they feel that they have a secure base (Ainsworth, 1979, p. 214). The finding that children played more during the video-link to their parent than the audio-link, further validated that video provides a stronger sense of proximity than audio, and is thus a more appropriate medium through which a parent can act as a secure base.

Our findings are also consistent with research that revealed that adults experience higher propinquity (i.e., closeness) when they interacted via a video-link than an audio-link (Walther and Bazarova, 2008). Although the current experiment objectively coded for behaviors that typically occur in securely attached children when their parent is physically proximal, the adults were self-reporting a comparable measure. Together, the findings of these two studies illustrate the superiority of video communication for adults and children alike.

We do acknowledge some minor but notable limitations to this research. One limitation is that all our participants were deemed to be securely attached to the participating parent – and that our experimental procedures were designed to elicit and assess the behavioral patterns typical of securely attached children. Thus, alternative experiential designs would need to be employed to assess the use of video communication with children with avoidant, ambivalent, and disorganized attachment styles. It should be noted that, although untested, we have no reason to assume that the advantages of video communication over audio with respect to parental propinquity would differ for children with insecure attachment styles.

Another limitation is that we are reporting on laboratory-based findings, and it is possible that behavioral and emotional patterns could differ in the home environment. Nonetheless, we believe that the findings of this research support the theorized value of video communication with young children for maintaining relationships. Similar research should eventually be undertaken examining video communication and audio communication in more ecologically valid scenarios.

Conversely, an additional limitation is that our design did not tightly constrain the adult contribution to the communication sessions. For example, parents may have communicated differently when communicating with their child via video than via audio-only. Thus, differences in the parental behavior across the separation sessions may have influenced their children’s behavior. While it would be interesting for future studies to evaluate the effects of parental behavior, our results are arguably more ecologically valid because parents were instructed to behave as they typically would during an audio or video communication session.

In summary, our findings demonstrate that compared with audio, video allows for more meaningful communication with young children. In addition to providing a sense of proximity and security more akin to physical presence than an audio-link, children were happier during the video-link than audio-link, which in a practical application would influence their motivation to participate. Given that video provides a stronger sense of proximity and engagement than audio, it follows that video communication is a more beneficial tool for maintaining family relationships with children during times of geographical separation.

The experiment described here considers a relatively new technology, and examines its appropriateness for young children compared to telephone communication. It highlights the importance of the visual elements of video communication for the child to obtain a sense of proximity with his or her parent. Given that extended families are increasingly separated by large distances, thus limiting traditional face-to-face contact (De Bruycker, 2008), understanding the effectiveness of communication tools for young children is of utmost importance. The evidence presented in this paper indicates that these video episodes may provide a “real enough” experience for children whereas audio alone is unable to provide the same effect. As such, future research should focus on video communication and its unrealized benefits to children. Moving forward, the practical application of video communication should be investigated in scenarios where children are separated from relatives for extended periods of time.

## Author Contributions

Conceived and designed the experiment: Jordy Kaufman, Joanne Tarasuik, Roslyn Galligan. Performed the experiment: Joanne Tarasuik. Analyzed the data: Joanne Tarasuik, Jordy Kaufman. Wrote the paper: Joanne Tarasuik, Jordy Kaufman, Roslyn Galligan.

## Conflict of Interest Statement

The authors declare that the research was conducted in the absence of any commercial or financial relationships that could be construed as a potential conflict of interest.
